# APH(3’)-Ie, an aminoglycoside-modifying enzyme discovered in a rabbit-derived *Citrobacter gillenii* isolate

**DOI:** 10.3389/fcimb.2024.1435123

**Published:** 2024-07-30

**Authors:** Naru Lin, Yuning Sha, Guozhi Zhang, Chunhan Song, Yuan Zhang, Jingxuan Zhao, Dawei Huang, Junwan Lu, Qiyu Bao, Wei Pan

**Affiliations:** ^1^ Institute of Bioinformatics, School of Laboratory Medicine and Life Sciences, Wenzhou Medical University, Wenzhou, China; ^2^ Medical Molecular Biology Laboratory, School of Medicine, Jinhua Polytechnic, Jinhua, China; ^3^ Department of Laboratory Sciences, The People’s Hospital of Yuhuan, Yuhuan, China; ^4^ Department of Laboratory Sciences, Pingyang Hospital of Wenzhou Medical University, Pingyang, China

**Keywords:** *Citrobacter gillenii*, resistance mechanism, aminoglycoside 3’phosphotransferase, *aph(3')-Ie*, kinetic parameter

## Abstract

**Background:**

Aminoglycoside-modifying enzymes (AMEs) play an essential role in bacterial resistance to aminoglycoside antimicrobials. With the development of sequencing techniques, more bacterial genomes have been sequenced, which has aided in the discovery of an increasing number of novel resistance mechanisms.

**Methods:**

The bacterial species was identified by 16S rRNA gene homology and average nucleotide identity (ANI) analyses. The minimum inhibitory concentration (MIC) of each antimicrobial was determined by the agar dilution method. The protein was expressed with the pCold I vector in *E. coli* BL21, and enzyme kinetic parameters were examined. The whole-genome sequence of the bacterium was obtained via the Illumina and PacBio sequencing platforms. Reconstruction of the phylogenetic tree, identification of conserved functional residues, and gene context analysis were performed using the corresponding bioinformatic techniques.

**Results:**

A novel aminoglycoside resistance gene, designated *aph(3’)-Ie*, which confers resistance to ribostamycin, kanamycin, sisomicin and paromomycin, was identified in the chromosome of the animal bacterium *Citrobacter gillenii* DW61, which exhibited a multidrug resistance phenotype. APH(3’)-Ie showed the highest amino acid identity of 74.90% with the functionally characterized enzyme APH(3’)-Ia. Enzyme kinetics analysis demonstrated that it had phosphorylation activity toward four aminoglycoside substrates, exhibiting the highest affinity (*K*_m_, 4.22 ± 0.88 µM) and the highest catalytic efficiency [*k*_cat_/*K*_m_, (32.27 ± 8.14) × 10^4^] for ribomycin. Similar to the other APH(3’) proteins, APH(3’)-Ie contained all the conserved functional sites of the APH family. The *aph(3’)-Ie* homologous genes were present in *C. gillenii* isolates from different sources, including some of clinical significance.

**Conclusion:**

In this work, a novel chromosomal aminoglycoside resistance gene, designated *aph(3’)-Ie*, conferring resistance to aminoglycoside antimicrobials, was identified in a rabbit isolate *C. gillenii* DW61. The elucidation of the novel resistance mechanism will aid in the effective treatment of infections caused by pathogens carrying such resistance genes.

## Introduction

Since the discovery and isolation of the first aminoglycoside antibiotic, streptomycin, from soil bacteria in 1944 (Jones et al., 1944), aminoglycoside antibiotics have been widely used as important anti-infection drugs (Zárate et al., 2018). They usually have good intrinsic activity against gram-negative and some gram-positive bacteria, killing them by binding to bacterial ribosomes and inhibiting normal protein synthesis (Kudo and Eguchi, 2016). Previous studies have shown that bacterial resistance to aminoglycoside antibiotics is usually driven by three main mechanisms: inactivation of the drug by aminoglycoside-modifying enzymes (AMEs), efflux, and targeted modification (e.g., by methylases) (Dozzo and Moser, 2010; Bastian et al., 2022).

Aminoglycoside *O*-phosphotransferases (APHs) are AMEs that utilize ATP or GTP to mediate the catalytic transfer of a phosphate group to an aminoglycoside molecule and make the antimicrobials inactivated. There are 35 functionally characterized APHs in the database CARD, and they are classified into 7 subfamilies [APH (2”), APH(3’), APH(3”), APH(4), APH(6’), APH(7”) and APH(9)] (Alcock et al., 2023). Located over chromosomes and plasmids, they are sometimes associated with mobile genetic elements (MGEs), such as transposons, integrons and conjugative elements, which made them spread between bacteria of different species or genera by means of horizontal gene transfer (Toth et al., 2010).

Including 21 species, *Citrobacter* is a genus of the family *Enterobacteriaceae*. The bacteria of this genus have been clinically reported to cause central nervous system infections and sepsis in neonates and immunocompromised hosts (Doran, 1999). The species *Citrobacter gillenii* (type strain ATCC 51117 = CCUG 30796 = CIP 106783 = DSM 13694) of the genus was characterized by DNA hybridization and biochemical analysis in 1999 (Brenner et al., 1999). In the recent years, it has been increasingly isolated from aquaculture animals, where it has been found to carry multidrug resistance (MDR) genes which became a challenge for the treatment of farmed animals (Duman et al., 2017; Concha et al., 2021; Türe et al., 2022).

In this study, based on the whole genome sequencing, a novel aminoglycoside *O*-phosphotransferase gene, designated *aph(3’)-Ie*, was identified in the chromosome of an animal isolate *C. gillenii* DW61, and its molecular and functional characteristics were further investigated.

## Materials and methods

### Bacteria and plasmids

*C. gillenii* DW61 was isolated from an anal swab of an *Oryctolagus cuniculus* f. *domesticus* from a farm in Wenzhou, Zhejiang Province, China. The anal swab sample was streaked onto a standard Luria–Bertani agar plate, and single colonies were then isolated and purified by the same method. Species classification was first performed by 16S rRNA gene homology comparison (Wachino and Arakawa, 2012) and then confirmed by average nucleotide identity (ANI) calculations (Lindsey et al., 2023) and DNA-DNA hybridization (isDDH) analysis (Meier-Kolthoff et al., 2014). The strains and plasmids used in this study are listed in **Table 1**.

**Table 1:** Strains and plasmids used in this work.

Strains or plasmids	Function	Reference
Strains		
DW61	The wild-type strain of *C. gillenii* DW61	This study
DH5α	*E. coli* DH5α was used as the host bacterium for cloning of the *aph(3’)-Ie* gene with its upstream promoter region	CGMCC^*^
ATCC 25922	*Pseudomonas aeruginosa* ATCC 25922 was used as a quality control strain for antimicrobial susceptibility testing	CGMCC^*^
DH5α(pMD19-T-*aph(3’)-Ie*)	*E. coli* DH5α carrying the recombinant plasmid pMD19-T- *aph(3’)-Ie*	This study
DH5α(pMD19-T)	*E. coli* DH5α carrying the pMD19-T vector was used as a control strain in the drug susceptibility testing	CGMCC^*^
BL21(pCold I-*aph(3’)-Ie*)	*E.coli* BL21 carrying the recombinant plasmid pCold I-*aph(3’)-Ie*	This study
Plasmids		
pMD19-T	T-Vector pMD™19 (Simple) was used as a vector for cloning of the *aph(3’)-Ie* gene with its upstream promoter region, AMP^r^	CGMCC^*^
pCold I	pCold I was used as a vector for expression of the ORF of the *aph(3’)-Ie* gene, AMP^r^	CGMCC^*^

*CGMCC, China General Microbiological Culture Collection Center.

### Drug susceptibility testing

The minimum inhibitory concentration (MIC) was tested by the plate dilution method using Mueller–Hinton (MH) agar (Thermo Fisher Scientific Inc. Beijing, China) in accordance with the most recent M100 performance standards for antimicrobial susceptibility testing by the Clinical and Laboratory Standards Institute (CLSI, 2024). After 16-20 h of constant incubation at 37°C, the results were interpreted following the CLSI M100 performance standards and the guidelines of the European Committee on Antimicrobial Susceptibility Testing (EUCAST, 2022). *Escherichia coli* ATCC 25922 was used for a quality control. The experiments were conducted in triplicate.

#### Genome sequencing, assembly, annotation, and bioinformatics analysis

The bacterial genomic DNA was extracted using the Universal Genomic DNA Purification Mini Spin Kit (Beyotime Biotechnology Co., Ltd., Shanghai, China). DNA sequencing was performed by using the Illumina NovaSeq 6000 and PacBio Sequel II platforms by Shanghai Personal Biotechnology Co., Ltd. (Shanghai, China). The Illumina short reads were assembled by SKESA v2.4.0 (Souvorov et al., 2018). The long reads from PacBio Sequel II were hybrid assembled with short reads in a short-read-first manner using Unicycler (v0.4.8) (Wick et al., 2017) and then polished by Pilon (v 1.23) (Walker et al., 2014). The prediction and annotation of the open reading frames (ORFs) were performed using prokka (Seemann, 2014) against UniProtKB/Swiss-Prot (http://web.expasy.org/docs/swiss-prot_guideline.html), and further annotation was performed by comparison with the NCBI nonredundant (nr) protein database using DIAMOND (v2.0.14) (Buchfink et al., 2021). MAFFT (v7.407) (Rozewicki et al., 2019) was used to perform multiple-sequence alignment. To construct a phylogenetic tree, IQ-TREE v2.2.2.3 (Minh et al., 2020b) was used to select the model that minimized the Bayesian information criterion (BIC) score, employing the log-likelihood method (Minh et al., 2020a). CD-search (Marchler-Bauer and Bryant, 2004) was used to predict the protein domains of APH(3’)-Ie by comparison with the Conserved Domain Database (CDD) (Wang et al., 2023). Clinker (v.0.0.25) (Gilchrist and Chooi, 2021) was used to perform genetic context analyses of *aph(3’)-Ie* and other related sequences.

#### Cloning of the *aph(3’)-Ie* gene

PCR was performed using primers to amplify the ORF of *aph(3’)-Ie* with its upstream promoter region predicted by BPROM (www.softberry.com) (**Table 2**). The PCR product was subsequently inserted into the T-Vector pMD™19 using T4 ligase (Takara Biomedical Technology Co., Ltd.). The recombinant plasmid in the ligation mixture was then transformed into competent *E. coli* DH5α cells by chemical transformation (Koppel et al., 2017). After screening colonies using an agar plate containing 100 μg/mL ampicillin, the sequence of the cloned fragment was verified by Sanger DNA sequencing (Shanghai Sunny Biotechnology Co., Ltd., Shanghai, China).

**Table 2:** Primers used in this study.

Primer^a^	Sequence (5’ → 3’)	Vector	Restriction endonuclease
F-61-clo	CGGGATCCGGTTCTCTTCTGCTCTAATCATC-ATTCAT	pMD19-T	*Bam*H I
R-61-clo	CGGAATTCGAGAGCAACTGTTCTTAGCCGT-TGT	pMD19-T	*Eco*R I
F-61-exp	GGAAGCTTCTGGTGCCGCGCGGCAGCAT-GAATTATATTCAAAGGGAAAAGCAGTGC-TCAGCT	pCold I	*Hind* III
R-61-exp	GCTCTAGACAACTGTTCTTAGCCGTTGT-AACCCTTTGAT	pCold I	*Xba* I

a Primers ending with “clo” were used to clone the ORF of the *aph(3’)-Ie* gene and its promoter region; primers ending with “exp” were used to clone the ORF of the *aph(3’)-Ie* gene. Restriction enzyme sites are underlined.

#### Expression and purification of APH(3’)-Ie

APH(3’)-Ie was expressed according to previously described methods with minor modifications (Qing et al., 2004; Sha et al., 2023). The ORF of the *aph(3’)-Ie* gene with the thrombin cleavage site was PCR-amplified (**Table 2**) and then ligated into the pCold I vector, and the recombinant plasmid pCold I-*aph(3’)-Ie* was subsequently transformed into *E. coli* BL21 (**Table 1**). To obtain APH(3’)-Ie, recombinant BL21(pColdI- *aph(3’)-Ie*) was grown to an OD_600_ of 0.5 in 100 mL of LB at 37°C. Then, 1 mM isopropyl-β-D-thiogalactopyranoside was added, and the mixture was incubated at 16°C for 16 h. Bacteria were collected by centrifugation at 10,000 × g for 4 min, resuspended in 4 mL of nondenatured lysate, and immediately sonicated for 10 min. The lysis products were subsequently centrifuged at 10,000 × g for 30 min at 4°C. The supernatant was collected and re-equilibrated with nickel-nitrilotriacetic acid (Ni-NTA) agarose resin (Beyotime Biotechnology, Shanghai, China). The mixture was gently agitated for 10 h at 4°C. Standard Ni-NTA affinity chromatography was used to isolate the His-tagged recombinant protein, and the His tag was removed using thrombin for 6 h at 37°C. The purified protein APH(3’)-Ie was validated using SDS-PAGE, and the protein concentration was determined with the BCA Protein Assay Kit (Beyotime Biotechnology, Shanghai, China).

### Enzyme kinetics analysis

The kinetic parameters of APH(3’)-Ie were measured in accordance with the previously reported methodology (Lu et al., 2021; Sha et al., 2023), with minor modifications. In brief, coupling between the production of ADP resulting from aminoglycoside phosphorylation and NADH oxidation was achieved through the use of pyruvate kinase (PK) and lactate dehydrogenase (LDH). The mixing volume for the entire reaction was 250 μL, which included 1 mM phosphoenolpyruvate (Beijing Solarbio Science & Technology Co., Ltd.), 600 μM NADH, 1 mM ATP, 100 mM HEPES (pH 7.0), 1 mM MgCl_2_, 2 mM KCl, a commercial mixture of PK and LD (Sigma P0294; 18-26 U/mL PK and 25-35 U/mL LDH, final concentrations), 30-40 nM APH(3′)-Ie and an aminoglycoside substrate at various concentrations. The ADP production rate was determined by monitoring the decrease in absorbance at 340 nm using a SpectraMax M5 multifunctional microplate reader (Molecular Devices, CA, United States) at 25°C. The experiments were conducted in triplicate. The velocities at steady state were determined by analyzing the linear phase of the reaction progress curve and then plotted as a function of the substrate concentration. The Michaelis-Menten equation was used to fit the data through nonlinear regression, with velocity as a function of substrate. GraphPad Prism 8.0.2 (GraphPad Software, CA, United States) was used for curve fitting and calculation of the dynamic parameters *K*_m_ and *k*_cat_.

#### Data availability

The nucleotide sequences of the *C. gillenii* DW61 genome have been submitted to GenBank under accession numbers CP151088 for the chromosome, CP151089 for the plasmid pDW61, and PP596862 for the *aph(3’)-Ie* gene.

## Results and discussion

### Identification of the novel resistance gene and classification and molecular characterization of the isolate DW61

To elucidate novel resistance mechanisms exhibited by bacterial isolates from local animals in response to antimicrobial agents, a total of 576 bacteria isolated from anal fecal swabs of domestic avians and livestock and environment in Wenzhou, China, were subjected to sequencing analysis (Feng et al., 2022; Gao et al., 2022; Zhao et al., 2023). Annotation of the genome data revealed the presence of resistance genes against various classes of antibiotics. Notably, among the predicted genes were some putative aminoglycoside antibiotic resistance genes, including but not limited to *aph(3’)-Ia*, *ant(9)-Ia*, *aadA5*, *aph(6)-Ic*, *aac(3)-IIIb*, *aac(6’)-Iaa*, *aph(6)-Id*, and *aac(2’)-IIb* homologues. These genes shared amino acid sequence identities below 80.0% with functionally characterized aminoglycoside resistance genes. Some of the genes were randomly selected and further cloned, and their resistance functions were determined. Ultimately, a novel *aph(3’)-Ia* homologous gene (designated *aph(3’)-Ie* in this work) that confers resistance to several aminoglycoside agents was identified from an isolate named DW61.

DW61 was isolated from the anal feces of a rabbit, and it showed high MICs of ≥ 16 µg/mL for 76.7% (33/43) of the antibiotics tested, particularly for aminoglycosides (**Table 3**). For the 10 aminoglycosides tested, except for amikacin, which had an MIC of 4 µg/mL, the MICs were ≥ 32 µg/mL. The MICs of ribostamycin, paromomycin, kanamycin and streptomycin were > 4096, > 2048, > 1024 and > 1024 µg/mL, respectively (**Table 3**).

**Table 3:** MIC results of the recombinants and control strains (µg/mL).

Class	Antibiotic	*C. gillenii* DW61	DH5α(pMD19-T-*aph(3’)-Ie*)	DH5α(pMD19-T)	DH5α	ATCC 25922
Aminoglycosides	Streptomycin	>1,024	2	2	2	4
	Ribostamycin	>4,096	512	<2	<2	4
	Gentamicin	128	0.5	0.5	0.25	0.5
	Tobramycin	256	0.5	0.5	0.5	0.5
	Kanamycin	>1,024	512	8	1	8
	Sisomicin	32	2	0.125	0.25	<1
	Amikacin	4	<2	1	1	2
	Netilmycin	32	<0.125	0.0625	0.0625	<0.125
	Paromomycin	>2,048	256	8	4	4
	Neomycin	128	16	8	16	1
Aminocyclitols	Spectinomycin	1,024	8	8	8	8
β-Lactams	Amoxicillin	2,048	/	/	/	1,024
	Piracillin	64	/	/	/	8
	Penicillin G	2,048	/	/	/	2,048
	Ampicillin	512	/	/	/	512
	Cefthiophene	256	/	/	/	>1,024
	Cefuroxime	16	/	/	/	512
	Cefazolin	64	/	/	/	>1,024
	Ceftriaxone	1	/	/	/	32
	Cefepime	0.125	/	/	/	0.5
	Cefoxitin	128	/	/	/	>512
	Cefotaxime	1	/	/	/	8
	Ceftazidime	8	/	/	/	1
	Aztreonam	0.5	/	/	/	2
	Imipenem	0.25	/	/	/	2
	Meropenem	<0.004	/	/	/	0.5
Quinolones	Enrofloxacin	16	/	/	/	/
	Levofloxacin	1	/	/	/	0.5
	Nalidixic acid	>1,024	/	/	/	128
Tetracycline	Tetracycline	>128	/	/	/	4
	Doxycycline	128	/	/	/	/
	Tigecycline	0.125	/	/	/	0.5
	Oxytetracycline	>64	/	/	/	/
Chloramphenicol	Chloramphenicol	128	/	/	/	128
	Florfenicol	256	/	/	/	64
Macrolides	Avermectin	258	/	/	/	/
	Tylosin	>1,024	/	/	/	/
	Acetylmequine	256	/	/	/	/
	Azithromycin	64	/	/	/	16
	Erythromycin	1,024	/	/	/	128
Lincoamide	Lincomycin	>1,024	/	/	/	/
	Sulfanilamide	256	/	/	/	/
Other antibiotics	Fosfomycin	1,024	/	/	/	8

“/” the test was not performed.

**Figure 1:**
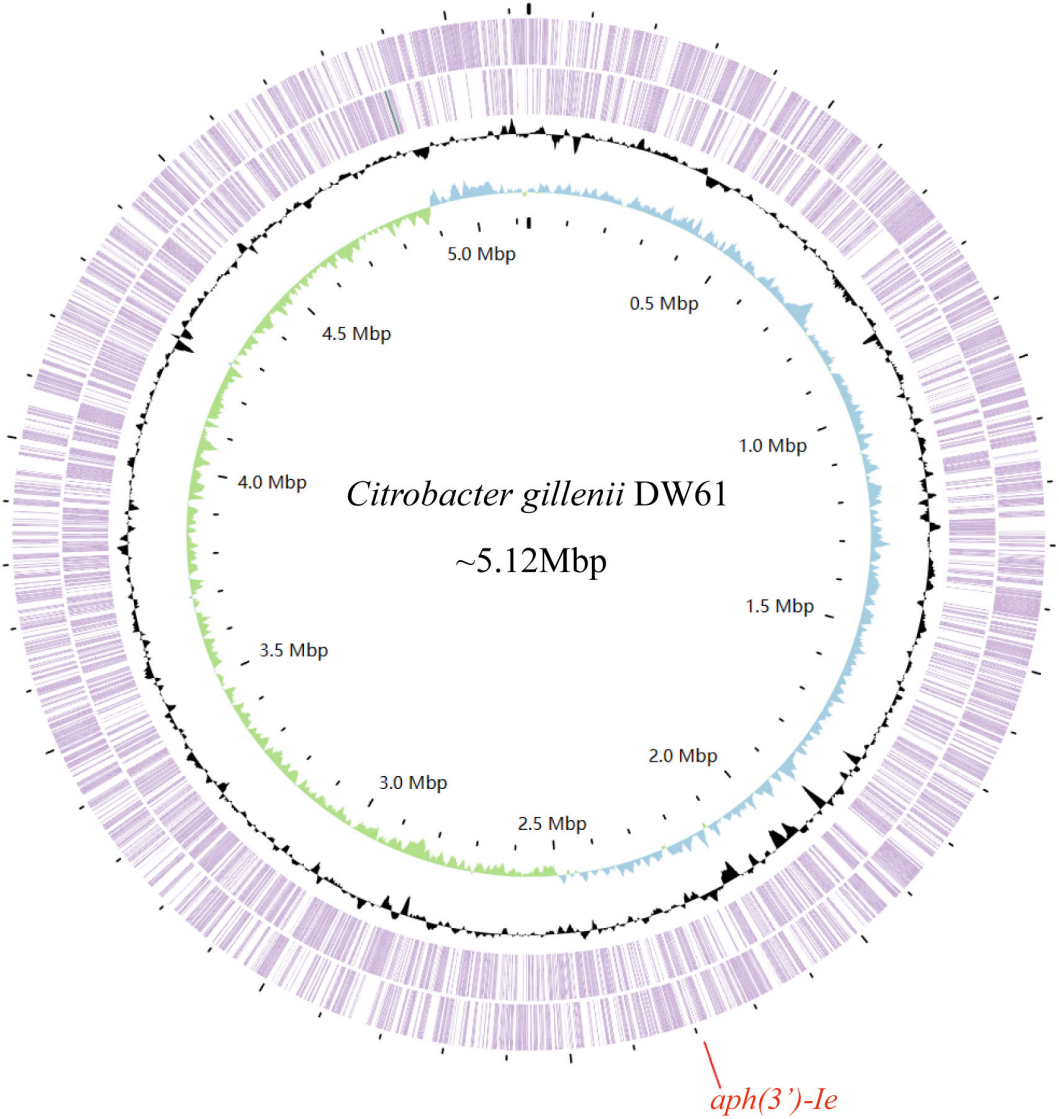
The genome map of *C. gillenii* DW61. The circles 1-4 from inside to outside represent the GC skew, GC content, and genes encoded in the forward and reverse strands of the chromosome of *C. gillenii* DW61, respectively. The red line on the bottom indicates the location of the novel resistance gene *aph(3’)-Ie*.

The genome of DW61 consists of a chromosome and a plasmid (designated pDW61-191). The chromosome is approximately 4.93 Mb in length and encodes 4,639 ORFs, and pDW61 is approximately 191.6 kb in length and encodes 237 ORFs (**Table 4**). 16S rRNA gene homology analysis revealed that the 16S rRNA gene of DW61 showed the highest identity (99.61%) with that of *C. gillenii* (AF025367.1). The genome-wide ANI analysis showed that the strain shared the highest identity (98.71%) with *C. gillenii* MBT-C3 (GCF_003429605.1) (**Figure 1**). Further DNA-DNA hybridization (DDH) indicated 87.80% identity between DW61 and *C. gillenii* MBT-C3 (GCF_003429605.1). This bacterium was thus classified as *C. gillenii* and designated *C. gillenii* DW61. The genome size of *C. gillenii* DW61 (5.13 Mb) was similar to that of three *C. gillenii* genomes (*C. gillenii* MBT-C3, GCA_003429605.1, 4.92 Mb; *C. gillenii* UMG736, GCA_013337685.1, 4.93 Mb; and *C. gillenii* AF64-5pH9A, GCA_027681665.1, 5.17 Mb) available in the NCBI genome database.

**Table 4:** General features of the DW61 genome.

	Chromosome	pDW61
Size (bp)	4,929,811	191,253
GC content (%)	52.45	52.50
ORFs	4,639	237
CDSs	4,536	237
Known proteins	3,356 (73.99%)	63 (26.6%)
Hypothetical proteins	1,180 (26.01%)	174 (73.4%)
Protein coding (%)	97.8	100
Average ORF length (bp)	946.5	717.6
Average protein length (aa)	318.5	238.2
tRNA	80	0
rRNA	(16S-23S-5S) × 7	0

### Functional analysis of the novel aminoglycoside 3’-phosphotransferase gene *aph(3’)-Ie*

The recombinant strain carrying *aph(3’)-Ie* showed >256-, 64-, 32-, 8-, and 2-fold greater MICs for ribostamycin, kanamycin, paromomycin, sisomicin, and neomycin, respectively, compared with the control strain (pMD19-T/DH5α). It did not show resistance to streptomycin, gentamicin, tobramycin, amikacin or netilmicin. This resistance phenotype is generally consistent with that associated with the *aph(3’)-I* class genes. The four close relatives of *aph(3’)-Ie* [*aph(3’)-Id*, *aph(3’)-Ib, aph(3’)-Ia* and *aphA15*] were previously reported to be resistant to kanamycin, neomycin, ribostamycin and paromomycin, similar to the resistance phenotype of *aph(3’)-Ie*. Moreover, these four *aph* genes also conferred susceptibility to gentamicin (except *aph(3’)-Ia*) or to amikacin (except aphA15) (Siregar et al., 1995; Riccio et al., 2001; Arcari et al., 2023; Sha et al., 2023).

**Figure 2:**
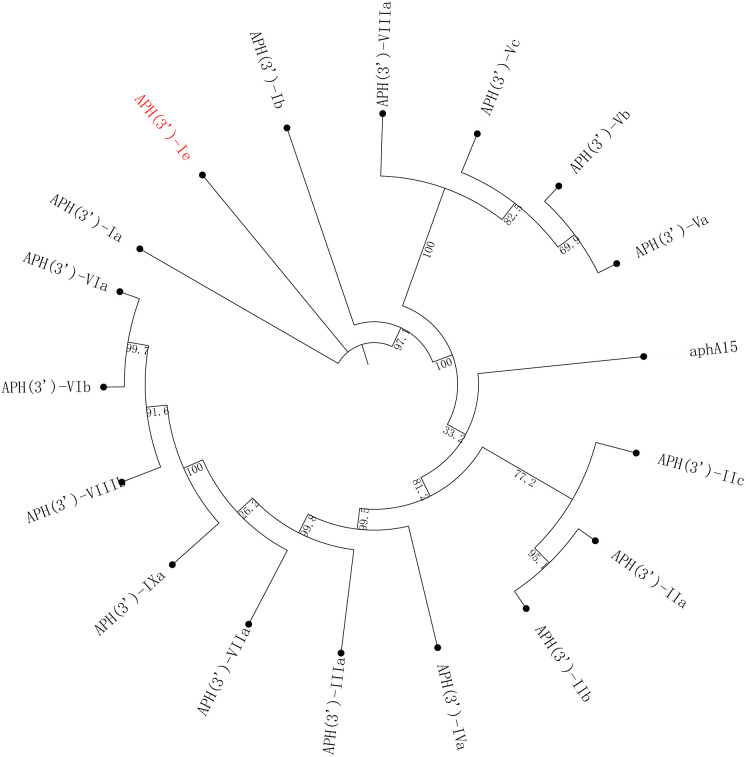
A phylogenetic tree showing the relationships of APH(3’)-Ie with other functionally characterized APH(3’)s. APH(3’)-Ie is highlighted in red. The accession numbers of these proteins are listed in **Supplementary Table S1**.

The *aph(3’)-Ie* gene is 816 bp in length and encodes a 271-amino-acid protein with a theoretical pI of 5.19. Notably, the comparison of APH(3’)-Ie with the functionally characterized proteins in the CARD database revealed that the proteins with the highest identities to the novel aminoglycoside 3’-phosphotransferase APH(3’)-Ie were APH(3’)-Ia (74.90%), APH(3’)-Ib (57.20%), APH(3’)-IIc (40.0%) and APH(3’)-IIa (35.97%). Phylogenetic analysis of APH(3’)-Ie with APH(3’)s revealed that it is also located closest to APH(3’)-Ia (**Figure 2**; **Supplementary Table S1**). This finding further confirmed that this protein is an aminoglycoside 3’-phosphotransferase (GenBank accession no. cd05150).

### Kinetic parameters and structural characterization of APH(3’)-Ie

The phosphotransferase activities of APH(3’)-Ie were generally consistent with the MIC results (**Table 5**). Among the six aminoglycoside substrates tested, APH(3’)-Ie exhibited phosphorylation activity toward ribomycin, kanamycin, neomycin and paromomycin but not gentamicin or streptomycin. It exhibited the highest affinity (*K*_m_, 4.22 ± 0.88 µM) and the highest catalytic efficiency [*k_cat_*/*K_m_*, (32.27 ± 8.14)^10^4^] for ribomycin.

**Table 5:** Kinetic parameters of APH(3’)-Ie.

Substrate	*k_cat_* (s^-1^)	*K_m_* (µM)	*k*_cat_/*K*_m_ (M^-1^/s^-1^)
Ribomycin	1.31 ± 0.09	4.22 ± 0.88	(3.23 ± 0.81)^10^5^
Kanamycin	1.06 ± 0.05	20.99 ± 1.84	(5.08 ± 0.44)^10^4^
Neomycin	1.65 ± 0.23	12.07 ± 0.40	(1.36 ± 0.13)^10^5^
Paromomycin	0.86 ± 0.01	7.74 ± 1.44	(1.14 ± 0.28)^10^5^
Streptomycin	NA	NA	NA
Gentamicin	NA	NA	NA

NA, no activity observed.

APH(3’)-Ia showed a similar affinity for kanamycin with APH(3’)-Ie (*K_m_*, 19.7 ± 2.5 vs. 20.99 ± 1.84 µM), but the catalytic efficiency of APH(3’)-Ia was much greater than that of APH(3’)-Ie [*k*_cat_/*K*_m_, 8.5 × 10^7^ vs. (5.08 ± 0.44) × 10^4^ M ^-1^/s^-1^], which was attributed to the much greater substrate transfer rate of APH(3’)-Ia for kanamycin than that of APH(3’)-Ie (*k*_cat_, 102 ± 14 vs. 1.06 ± 0.05 s^-1^) (Siregar et al., 1995). In contrast to APH(3’)-Ia, APH(3’)-IIa showed lower affinities for kanamycin and neomycin than did APH(3’)-Ie, with *K_m_* values of 3 vs. 20.99 ± 1.84 µM for kanamycin and 6 vs. 12.07 ± 0.40 µM for neomycin, respectively, but its catalytic efficiencies for both substrates were greater than those of APH(3’)-Ie [with *k*_cat_/*K*_m_ of 1.3 × 10^6^ vs. (5.08 ± 0.44) × 10^4^ M ^-1^/s^-1^ for kanamycin and 1.8 × 10^6^ vs. (1.36 ± 0.13) × 10^5^ M ^-1^/s^-1^ for neomycin, respectively]. This was also because the substrate transfer rates of APH(3’)-IIa for these two substrates were greater than those of APH(3’)-Ie (with *k_cat_* of 4 vs. 1.06 ± 0.05 s^-1^ for kanamycin and 11 vs. 1.65 ± 0.23 s^-1^ for neomycin, respectively) (Siregar et al., 1994).

**Figure 3:**
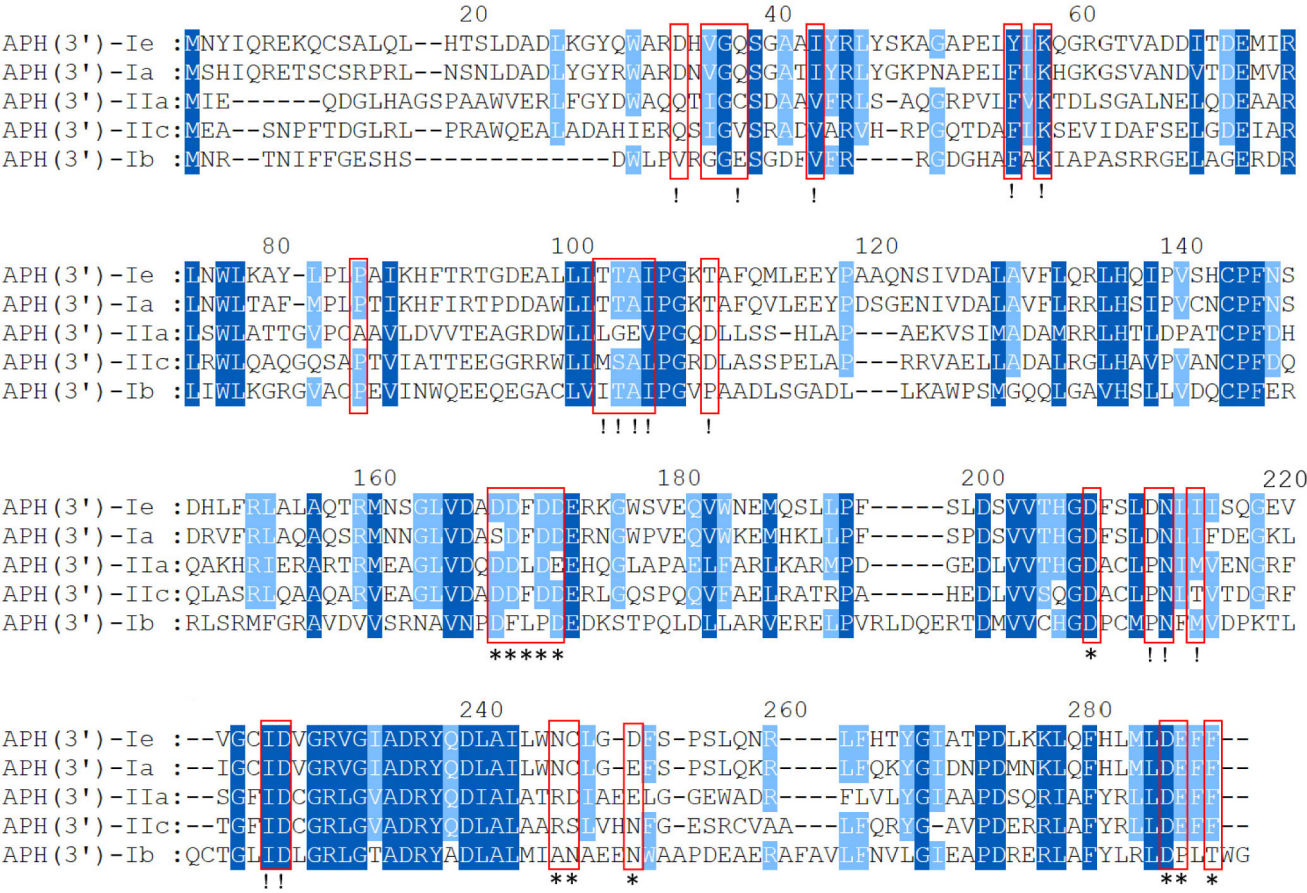
Multiple-sequence alignment of APH(3’)-Ie with its close relatives. The numbers on the right represent the corresponding amino acid sequence lengths. The red frames represent the residues of the conserved APH protein domain family. The exclamation marks indicate the residues of the ATP-binding sites, and the asterisks indicate the 12 residues of the antibiotic-binding site. The conserved residues are shown in dark blue, while the relatively conserved residues are shown in light blue. Gaps are represented using hyphens.

To analyze the structural conservation of the protein, the amino acid sequences of 5 APH(3’) proteins (including the one from this study) with close evolutionary relationships were compared. APH(3’)-Ie contained all 30 active residues of the conserved APH protein domain family (GenBank accession no. cd05150), including 15 residues of the ATP-binding site and 12 residues of the antibiotic-binding site (Fong and Berghuis, 2002; Nurizzo et al., 2003; Stogios et al., 2013) (**Figure 3**).

### Distribution of the *aph(3’)-Ie* genes and structural characterization of the *aph(3’)-Ie*-related sequences

**Figure 4:**
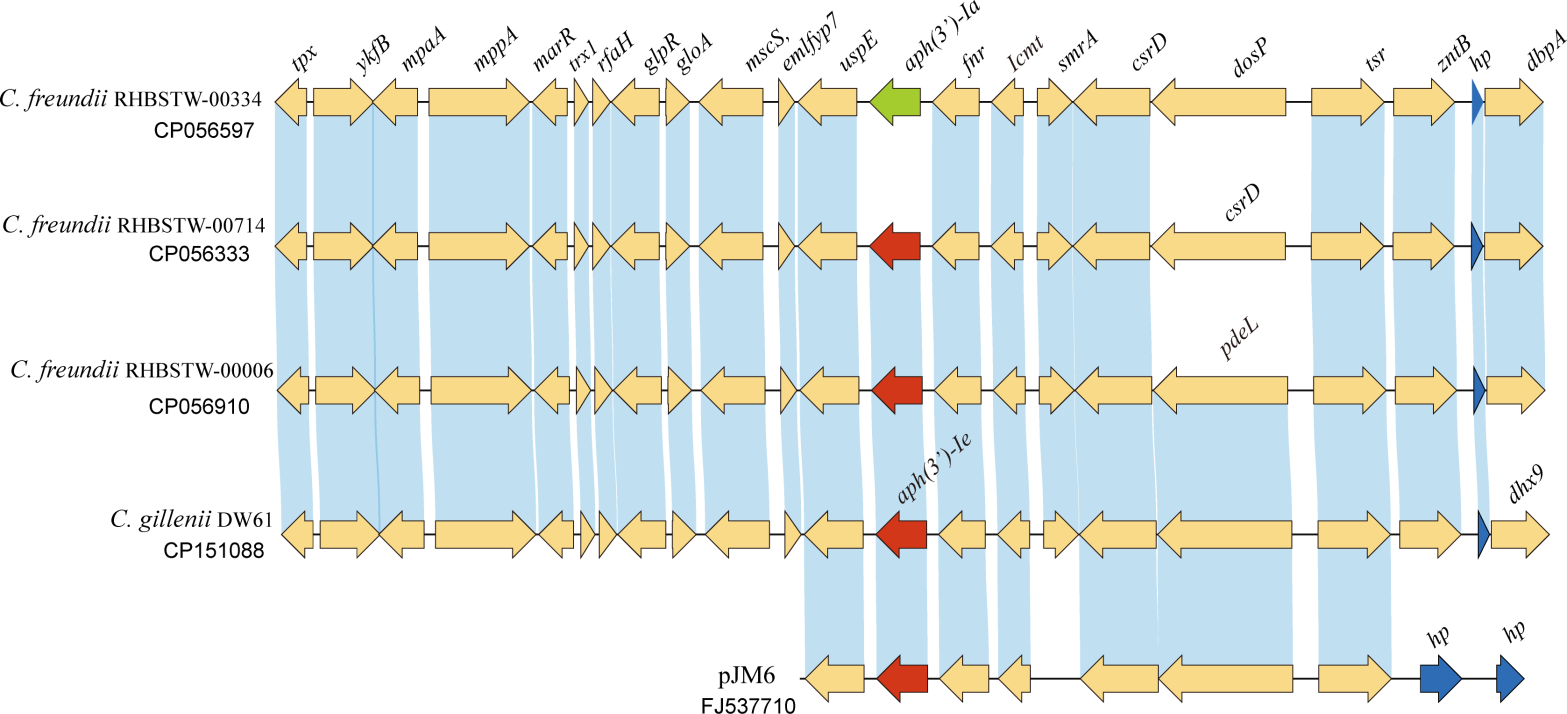
Genetic background of the *aph(3’)-Ie* gene and the *aph(3’)-Ie* homologous genes. Regions with an amino acid identity of ≥ 80% are in blue. hp, hypothetical protein. The *aph(3’)-Ie* and *aph(3’)-Ie*-like genes (similarity > 98.15%) are shown in red. The *aph(3’)-Ia* gene is shown in green, and the hypothetical protein-encoding genes are shown in blue.

To analyze the distribution of the *aph(3’)-Ie* gene and its close relatives, the amino acid sequence of APH(3’)-Ie was used as a query to search for homologous protein-encoding nucleotide sequences using the tBLASTn program against the NCBI nucleotide collection (nt) database (**Figure 4**), and a total of 28 sequences showing ≥ 98.15% amino acid sequence identity with APH(3’)-Ie were found in the database. The three strains with the highest identities (100.0%) were all *C. freundii* strains (including *C. freundii* RHBSTW-00714, CP056333.1; *C. freundii* RHBSTW-00006, CP056910.1; and *C. freundii* RHBSTW-00334, CP056597.1). Among the remaining 25 sequences, one was from the uncultured bacterium pJM6 from a metagenomic library of an environmental sample (FJ537710.1, identity 98.89%) and one was from *Citrobacter werkmanii* NBRC 105721 (LR699014.1, identity 98.15%), while the other 23 genes were also from *C. freundii*. Of the 28 genes, one was from a metagenomic library of an environmental sample, while the other 27 were from strains isolated from different sources, including animals (92.59%, 25/27), the environment (3.70%, 1/27) and humans (3.70%, 1/27) (**Supplementary Table S2**).

Further analysis of the sequences with lower identities revealed that of the 100 sequences with identities ≥ 70.94%, which included the 28 sequences mentioned above, 72 sequences had identities between 74.91% and 70.94%, and no sequence with an identity between > 74.91% and < 98.15% was present. Notably, of these 72 sequences with identities ranging from 70.94% to 74.91%, three sequences were from *Klebsiella pneumoniae*, including two encoded on plasmids (pBWH77 of *K. pneumoniae* 5214773, NG_047431.1, and pBWH77 of *K. pneumoniae*, X62115.1) and one in the chromosome (*K. pneumoniae* GDKA1, NG_047441.1), while the others were from other orders of bacteria, cloning vectors or synthetic constructs (**Supplementary Table S2**).

When analyzing the structure of the *aph(3’)-Ie* gene-related sequences, a nucleotide sequence approximately 20 kb in length with the *aph(3’)-Ie* gene at the center from the *C. gillenii* DW61 chromosome was used as a query to search for homologous sequences in the NCBI nucleotide database. A total of 28 sequences with identities ≥ 80.0% were retrieved, and they were all from the genus *Citrobacter*. Of these 28 sequences, 27 carried one *aph(3’)-Ie*(-like) gene each and were from *C. freundii*, except one from *C. werkmanii*, MGYG-HGUT-02535 (LR699014.1, identity 98.41%). One sequence free of an *aph(3’)-Ie*-like gene was from another species, *Citrobacter ructae* SNUWT2 (CP038469.1), and it shared a lower identity (81.62%) with that of *C. gillenii* DW61. No mobile genetic elements (MGEs) were detected within these 20 kb fragments.

## Conclusion

In this study, a novel aminoglycoside phosphotransferase gene, designated *aph(3’)-Ie*, was identified in the chromosome of the MDR *Citrobacter gillenii* isolate DW61, which showed high MICs (≥ 16 μg/mL) for 76.7% of the 43 antibiotics tested. This novel resistance gene conferred resistance to some aminoglycosides, such as ribostamycin and kanamycin, and was present in *C. gillenii* from different sources, including some of clinical significance. The discovery of new antimicrobial resistance mechanisms is helpful for effective clinical treatment of bacterial infectious diseases.

## Data availability statement

The datasets presented in this study can be found in online repositories. The names of the repository/repositories and accession number(s) can be found in the article/**Supplementary Material**.

## Ethics statement

This study used strains isolated from animals in animal farms in Wenzhou, China. The owners of the farms were informed in writing of the study and provided approval for the sampling of animals. The studies involving human participants and animals were reviewed and approved by the Animal Welfare and Ethics Committee of Wenzhou Medical University, Zhejiang Province, China (protocol number: wydw2021-0323).

## Author contributions

NL: Data curation, Formal analysis, Investigation, Methodology, Software, Validation, Writing – original draft. YS: Writing – original draft. GZ: Writing – original draft. CS: Writing – original draft. YZ: Writing – original draft. JZ: Writing – original draft. DH: Writing – original draft. JL: Writing – original draft. QB: Conceptualization, Data curation, Formal analysis, Funding acquisition, Investigation, Methodology, Project administration, Resources, Software, Supervision, Validation, Visualization, Writing – original draft, Writing – review & editing. WP: Writing – original draft, Writing – review & editing.

## Supplementary material

Supplementary Table 1 Proteins used for phylogenetic tree construction.

Supplementary Table 2 Distribution of *aph(3’)-Ie*(-like) genes.
