# Supplementary material for: APH(3’)-Ie, an aminoglycoside-modifying enzyme discovered in a rabbit-derived Citrobacter gillenii isolate
Source: Front Cell Infect Microbiol. 2024 Jul 30;14:1435123. doi: 10.3389/fcimb.2024.1435123 (PMC11320999; doi:10.3389/fcimb.2024.1435123)
Supplement: Supplementary Table 1 — Proteins used for phylogenetic tree construction. [file Table_1.docx]

Table S1 Proteins used for phylogenetic tree construction.

| Protein | ARO Accession No. | Protein Accession No. | DNA Accession No. |
| --- | --- | --- | --- |
| APH(3')-Ia | ARO:3002641 | CAE51638.1 | BX664015.1 |
| APH(3')-Ib | ARO:3002642 | AAA26412.1 | M20305.1 |
| APH(3')-IIa | ARO:3002644 | CAA23892.1 | V00618.1 |
| APH(3')-IIb | ARO:3002645 | CAA62365.1 | X90856.1 |
| APH(3')-IIc | ARO:3002646 | ADQ43421.1 | HQ424460.1 |
| APH(3')-IIIa | ARO:3002647 | AGV10830.1 | CP004067.1 |
| APH(3')-IVa | ARO:3002648 | CAA27061.1 | X03364.1 |
| APH(3')-IXa | ARO:3004087 | ENV34035.1 | APPN01000061.1 |
| APH(3')-Va | ARO:3002649 | AAA26699.1 | K00432.1 |
| APH(3')-Vb | ARO:3002650 | AAC32025.1 | M22126.1 |
| APH(3')-Vc | ARO:3002651 | AAB21326.1 | S81599.1 |
| APH(3')-VIa | ARO:3002652 | CAA30578.1 | X07753.1 |
| APH(3')-VIb | ARO:3002653 | CAF29483.1 | AJ627643.4 |
| APH(3')-VIIa | ARO:3002654 | AAA76822.1 | M29953.1 |
| APH(3')-VIIIa | ARO:3004680 | AAG11411.2 | AF182845.2 |
| APH(3')-VIIIb | ARO:3004086 | EPF73263.1 | ATGI01000028.1 |
| APHA15 | ARO:3004675 | CAD91341.1 | Y18050.2 |
