# Supplementary material for: APH(3’)-Ie, an aminoglycoside-modifying enzyme discovered in a rabbit-derived Citrobacter gillenii isolate
Source: Front Cell Infect Microbiol. 2024 Jul 30;14:1435123. doi: 10.3389/fcimb.2024.1435123 (PMC11320999; doi:10.3389/fcimb.2024.1435123)
Supplement: Supplementary Table 2 — Distribution of aph(3’)-Ie(-like) genes. [file Table_2.docx]

Table S2 Distribution of *aph(3’)-Ie*(-like) genes.

| Name | Species | Accession | Identity (%) | Source |
| --- | --- | --- | --- | --- |
| *Citrobacter freundii* strain RHBSTW-00714 | *Citrobacter freundii* | CP056333.1 | 100 | Livestock host |
| *Citrobacter freundii* strain RHBSTW-00006 | *Citrobacter freundii* | CP056910.1 | 100 | Livestock host |
| *Citrobacter freundii strain RHBSTW-00334* | *Citrobacter freundii* | CP056597.1 | 100 | Livestock host |
| *Citrobacter freundii* strain RHB24-C03 | *Citrobacter freundii* | CP057509.1 | 99.63 | Livestock host |
| *Citrobacter freundii* strain RHB24-C05 | *Citrobacter freundii* | CP057506.1 | 99.63 | Livestock host |
| *Citrobacter freundii* strain RHBSTW-00968 | *Citrobacter freundii* | CP056229.1 | 99.26 | Livestock host |
| *Citrobacter freundii* strain RHBSTW-00408 | *Citrobacter freundii* | CP056559.1 | 99.26 | Livestock host |
| *Citrobacter freundii* strain RHBSTW-00923 | *Citrobacter freundii* | CP056244.1 | 99.26 | Livestock host |
| *Citrobacter freundii* strain RHBSTW-00398 | *Citrobacter freundii* | CP055538.1 | 98.89 | Livestock host |
| *Citrobacter freundii* strain Upstream_1 | *Citrobacter freundii* | CP038856.1 | 98.89 | Freshwater biome |
| Uncultured bacterium pJM6 unknown genes | uncultured bacterium pJM6 | FJ537710.1 | 98.89 | Environment |
| *Citrobacter freundii* strain RHB41-C16 | *Citrobacter freundii* | CP056967.1 | 98.89 | Livestock host |
| *Citrobacter freundii* strain RHB29-C07 | *Citrobacter freundii* | CP057353.1 | 98.52 | Livestock host |
| *Citrobacter freundii* strain RHBSTW-00880 | *Citrobacter freundii* | CP055906.1 | 98.52 | Livestock host |
| *Citrobacter freundii* strain RHB31-C16 | *Citrobacter freundii* | CP057258.1 | 98.52 | Livestock host |
| *Citrobacter freundii* strain RHBSTW-00477 | *Citrobacter freundii* | CP056473.1 | 98.52 | Livestock host |
| *Citrobacter freundii* strain RHB09-E3-C07 | *Citrobacter freundii* | CP099254.1 | 98.52 | Livestock host |
| *Citrobacter freundii* strain RHB36-C06 | *Citrobacter freundii* | CP057136.1 | 98.52 | Livestock host |
| *Citrobacter freundii* strain RHBSTW-00120 | *Citrobacter freundii* | CP056850.1 | 98.52 | Livestock host |
| *Citrobacter freundii* strain RHBSTW-00444 | *Citrobacter freundii* | CP056515.1 | 98.52 | Livestock host |
| *Citrobacter freundii* strain RHBSTW-00488 | *Citrobacter freundii* | CP055466.1 | 98.52 | Livestock host |
| *Citrobacter freundii* strain RHB13-SO-C05 | *Citrobacter freundii* | CP099222.1 | 98.15 | Livestock host |
| *Citrobacter freundii* strain RHBSTW-00267 | *Citrobacter freundii* | CP056644.1 | 98.15 | Livestock host |
| *Citrobacter werkmanii* isolate MGYG-HGUT-02535 | *Citrobacter werkmanii* | LR699014.1 | 98.15 | Human gut |
| *Citrobacter freundii* strain RHBSTW-00589 | *Citrobacter freundii* | CP056395.1 | 98.15 | Livestock host |
| *Citrobacter freundii* strain RHB43-C17 | *Citrobacter freundii* | CP099084.1 | 98.15 | Livestock host |
| *Citrobacter freundii* strain RHBSTW-00269 | *Citrobacter freundii* | CP055543.1 | 98.15 | Livestock host |
| *Citrobacter freundii* strain RHBSTW-00355 | *Citrobacter freundii* | CP056586.1 | 98.15 | Livestock host |
| pSe-Kan aminoglycoside phosphotransferase (aph) gene | S*almonella enterica* subsp. enterica serovar Typhimurium | GQ426885.1 | 74.91 |  |
| Aminoglycoside O-phosphotransferase APH(3')-Ia | *Klebsiella pneumoniae* | NG_047431.1 | 74.91 |  |
| *Acinetobacter baumannii* strain A071 transposon Tn6020b-1 | *Acinetobacter baumannii* | KT317080.1 | 74.91 |  |
| *Acinetobacter baumannii* strain A076 transposon Tn6020a-3 | *Acinetobacter baumannii* | KT317084.1 | 74.91 |  |
| *Acinetobacter baumannii* strain A072 transposon Tn6020a-1 | *Acinetobacter baumannii* | KT317082.1 | 74.91 |  |
| *Providencia stuartii* strain PsB/3 plasmid IncA/C class I integron | *Providencia stuartii* | JN193568.1 | 74.91 |  |
| *Acinetobacter baumannii* strain A070 transposon Tn6020a-2 | *Acinetobacter baumannii* | KT317083.1 | 74.91 |  |
| *K.pneumoniae* plasmid pBWH77 aphA7 and blaS2A genes | *Klebsiella pneumoniae* | X62115.1 | 74.91 |  |
| *Acinetobacter baumannii* strain A94 transposon Tn6020 | *Acinetobacter baumannii* | JF343535.1 | 74.91 |  |
| Aminoglycoside 3'-phosphotransferase-like protein gene, complete cds | *Acinetobacter baumannii* | JF519620.1 | 74.54 |  |
| Aminoglycoside O-phosphotransferase APH(3')-Ia | *Mannheimia haemolytica* | NG_056047.1 | 74.54 |  |
| Aminoglycoside O-phosphotransferase APH(3')-Ia | *Escherichia coli* | NG_047429.1 | 74.54 |  |
| *E.coli* aminoglycoside 3'-phosphotransferase gene | *Escherichia coli* | Y00452.1 | 74.54 |  |
| Aminoglycoside 3'-O-phosphotransferase (aph(3')-Id) gene | *Kluyvera intermedia* | OQ819314.1 | 74.54 |  |
| *Typhimurium* plasmid pST3553 aphA7 gene for aminoglycoside phosphotransferase | *Salmonella enterica subsp. enterica serovar Typhimurium* | AB571792.1 | 74.17 |  |
| Synthetic construct kanamycin resistance protein (kanR) gene | Synthetic construct | EU496093.1 | 74.17 |  |
| Aminoglycoside *O*-phosphotransferase APH(3')-Ia | *Escherichia coli* | NG_047430.1 | 74.17 |  |
| aph(3')-Ia gene for aminoglycoside *O-*phosphotransferase APH(3')-Ia | *Avibacterium paragallinarum* | NG_047439.1 | 74.17 |  |
| *Escherichia coli* plasmid p5 DNA | *Escherichia coli* | LC318101.1 | 74.17 |  |
| *Escherichia coli* strain RHB31-C08 plasmid | *Escherichia coli* | CP055666.1 | 74.17 |  |
| Aminoglycoside O-phosphotransferase APH(3')-Ia | *Enterobacter cloacae* | NG_047440.1 | 74.17 |  |
| Aminoglycoside O-phosphotransferase APH(3')-Ia | *Klebsiella pneumoniae* | NG_047441.1 | 74.17 |  |
| Aminoglycoside O-phosphotransferase APH(3')-Ia | *Proteus mirabilis* | NG_047438.1 | 74.17 |  |
| Aminoglycoside phosphotransferase gene | Synthetic construct | U63147.1 | 74.17 |  |
| Vector pGE | Vector pGE | MZ361922.1 | 74.17 |  |
| Transposon delivery vector pUTKm DNA | Transposon delivery vector pUTKm | AB158755.1 | 74.17 |  |
| *Escherichia coli* plasmid pV001-b | *Escherichia coli* | LC056095.1 | 74.17 |  |
| Cloning vector BASIC_2_KAN-pMB1 | Cloning vector BASIC_2_KAN-pMB1 | KP223696.1 | 74.17 |  |
| Vector pGEC | Vector pGEC | MZ361915.1 | 74.17 |  |
| *Escherichia coli* plasmid pV004-b DNA | *Escherichia coli* | LC056157.1 | 74.17 |  |
| Integrative expression vector pSyFPN | Integrative expression vector pSyFPN | JN698883.1 | 74.17 |  |
| Cloning vector pSEVA211 | Cloning vector pSEVA211 | JX560326.2 | 74.17 |  |
| Synthetic transposon TyK'GFP+ | Synthetic construct | U84737.1 | 74.17 |  |
| Synthetic construct mini-Tn5 KpF | Synthetic construct | JQ406586.1 | 74.17 |  |
| *Babesia bigemina* genome assembly Bbig001 | *Babesia bigemina* | LK054930.1 | 74.17 |  |
| Aminoglycoside 3'-phosphotransferase | Synthetic construct | FN394965.2 | 74.17 |  |
| Cloning vector pKF299 DNA | Cloning vector pKF299 | D63843.1 | 74.17 |  |
| Cloning vector pKF298 DNA | Cloning vector pKF298 | D63842.1 | 74.17 |  |
| Cloning vector pKF297 DNA | Cloning vector pKF297 | D63841.1 | 74.17 |  |
| Cloning vector pKF296 DNA | Cloning vector pKF296 | D63840.1 | 74.17 |  |
| Vector pMM1-30A | Vector pMM1-30A | KX981578.1 | 74.17 |  |
| *Vibrio* phage CTX transgenic isolate recombinant CTX2 Kan Zot (zot) gene | Affertcholeramvirus CTXphi | KF664576.1 | 74.17 |  |
| Cloning vector pKF4 DNA | Cloning vector pKF4 | D45833.1 | 74.17 |  |
| Expression vector pHsh-kan | Expression vector pHsh-kan | FJ571621.1 | 74.17 |  |
| Cloning vector pCDS | Cloning vector pCDS | MK873086.1 | 74.17 |  |
| Synthetic construct plasmid pGEpyrF | synthetic construct | MN450165.1 | 74.17 |  |
| Synthetic construct plasmid pGEung | synthetic construct | MN450166.1 | 74.17 |  |
| Cloning vector pSEVA212S | Cloning vector pSEVA212S | JX560376.2 | 74.17 |  |
| Synthetic construct plasmid pGEnfi | Synthetic construct | MN450167.1 | 74.17 |  |
| Uncultured bacterium clone PI_9F_Contig_3 genomic sequence | Uncultured bacterium | KU547746.1 | 74.17 |  |
| Cloning vector pY71-sfGFP | Cloning vector pY71-sfGFP | MT346027.1 | 74.17 |  |
| PM7 Kan Zot (zot) gene; Kan (kan) gene; and CtxB (ctxB) gene | Affertcholeramvirus CTXphi | KF664573.1 | 74.17 |  |
| Aminoglycoside *O*-phosphotransferase APH(3')-Ia | *Proteus vulgaris* | NG_047436.1 | 73.8 |  |
| Aminoglycoside *O*-phosphotransferase APH(3')-Ia | *Escherichia coli* | NG_047437.1 | 73.8 |  |
| Aminoglycoside 3'-phosphotransferase | Cloning vector pEN-Km | EF526064.1 | 73.8 |  |
| Plasmid Rst1 Tsg fragment ORF-I, and kanamycin resistance gene | Plasmid Rts1 | M65202.1 | 73.8 |  |
| Cloning vector pMA_Auxiliray_4D | Cloning vector pMA_Auxiliray_4D | MG252996.1 | 73.8 |  |
| Cloning vector pMA_Auxiliray_4C | Cloning vector pMA_Auxiliray_4C | MG252995.1 | 73.8 |  |
| Cloning vector pMA_Auxiliray_4B | Cloning vector pMA_Auxiliray_4B | MG252994.1 | 73.8 |  |
| Cloning vector pMA_Auxiliray_4A | Cloning vector pMA_Auxiliray_4A | MG252993.1 | 73.8 |  |
| Cloning vector pMA_Auxiliray_3 | Cloning vector pMA_Auxiliray_3 | MG252992.1 | 73.8 |  |
| Synthetic construct kanamycin kinase (aph) gene | synthetic construct | MW473472.1 | 73.43 |  |
| Cloning vector pSMART-HCKan | Cloning vector pSMART-HCKan | AF532107.1 | 73.43 |  |
| Cloning vector pSMARTGC HK | Cloning vector pSMARTGC HK | EU729724.1 | 73.43 |  |
| Cloning vector pSMART-LCKan | Cloning vector pSMART-LCKan | AF532106.2 | 73.43 |  |
| Cloning vector pSMARTGC LK | Cloning vector pSMARTGC LK | EU729725.2 | 73.43 |  |
| Cloning vector pEZSeq-Kan | Cloning vector pEZSeq-Kan | AF532108.1 | 73.43 |  |
| Synthetic construct Golden Gate selectable marker insert KanR sequence | Synthetic construct | OK165507.1 | 73.06 |  |
| *aph(3')-Ia* gene for aminoglycoside *O*-phosphotransferase APH(3')-Ia | *Salmonella enterica* subsp. enterica serovar Typhimurium | NG_047435.1 | 72.69 |  |
| Aminoglycoside *O*-phosphotransferase (G418) gene | Synthetic construct | MF116006.1 | 71.5694 |  |
| Aminoglycoside *O*-phosphotransferase APH(3')-Ia | Plasmid pFA6 | NG_047432.1 | 71.5694 |  |
| Aminoglycoside 3'-phosphotransferase | *Entamoeba histolytica* HM-1:IMSS | XM_001914605.1 | 70.9404 |  |
